# Supplementary material for: Influenza Vaccination of Nurses and Other Health Care Workers in Different Occupational Settings: A Classic and AI Mixed Approach for Time-to-Event Data
Source: Nurs Rep. 2025 Mar 3;15(3):87. doi: 10.3390/nursrep15030087 (PMC11944323; doi:10.3390/nursrep15030087)
Supplement: Supplementary file 1 [file nursrep-15-00087-s001.zip › SuppMat_S1.pdf]

# SUPPLEMENTARY MATERIALS S1

Article

Influenza Vaccination of Nurses and Other Health Care Workers in Different Occupational Settings: a Classic and AI Mixed Approach for Time-to Event Data

Matteo Ratti, Riccardo Rescinito, Domenico Gigante, Alberto Lontano, and Massimiliano Panella

# KAPLAN MEIER TABLES

Area Critica = Critical Care Unit

Chirurgico = Surgical Unit

Medico = Medical Unit

Servizi = Service Unit

Inferm. = Nurse

Prof. San. = Other HCW

CDC_GRUPPO = variable concerning the ward typology

QUALIF_GRUPPO = variable concerning the HCW tipology

DIM_ANNO = year variable

Table S1-1 – Kaplan Meier integral table

2024-12-21

```
load("my_work_space.RData")
tables_
```

```
## Call: survfit(formula = Surv(TIME_F, STATUS_F) ~ CDC_GRUPPO + 
QUALIF_GRUPPO, 
##     data = db)
## 
##                  CDC_GRUPPO=Area Critica, QUALIF_GRUPPO=Inferm. 
##  time n.risk n.event survival std.err lower 95% CI upper 95% CI
##     1    648       2    0.997 0.00218        0.993        1.000
##     4    646       1    0.995 0.00267        0.990        1.000
##     8    645       2    0.992 0.00344        0.986        0.999
```

```
##     9    643        1    0.991 0.00376        0.983        0.998
##    11    642        3    0.986 0.00460        0.977        0.995
##    14    639        7    0.975 0.00610        0.963        0.987
##    15    632        2    0.972 0.00646        0.960        0.985
##    17    630        3    0.968 0.00696        0.954        0.981
##    18    627        3    0.963 0.00742        0.949        0.978
##    21    624        4    0.957 0.00799        0.941        0.973
##    22    620        6    0.948 0.00876        0.931        0.965
##    23    614        4    0.941 0.00923        0.923        0.960
##    24    610        6    0.932 0.00988        0.913        0.952
##    25    604        7    0.921 0.01058        0.901        0.942
##    28    597        1    0.920 0.01067        0.899        0.941
##    30    596        3    0.915 0.01095        0.894        0.937
##    31    593        2    0.912 0.01113        0.890        0.934
##    36    591        2    0.909 0.01130        0.887        0.931
##    37    589        4    0.903 0.01164        0.880        0.926
##    38    585        7    0.892 0.01219        0.868        0.916
##    39    578        2    0.889 0.01235        0.865        0.913
##    40    576        2    0.886 0.01249        0.862        0.911
##    43    574        2    0.883 0.01264        0.858        0.908
##    44    572        2    0.880 0.01278        0.855        0.905
##    45    570        4    0.873 0.01306        0.848        0.899
##    46    566        2    0.870 0.01320        0.845        0.897
##    47    564        1    0.869 0.01326        0.843        0.895
##    49    563        1    0.867 0.01333        0.842        0.894
## 
##                 CDC_GRUPPO=Area Critica, QUALIF_GRUPPO=Prof. San. 
##  time n.risk n.event survival std.err lower 95% CI upper 95% CI
##    14     78       1    0.987  0.0127        0.963        1.000
##    17     77       1    0.974  0.0179        0.940        1.000
##    22     76       1    0.962  0.0218        0.920        1.000
##    23     75       1    0.949  0.0250        0.901        0.999
##    29     74       1    0.936  0.0277        0.883        0.992
##    32     73       1    0.923  0.0302        0.866        0.984
##    40     72       1    0.910  0.0324        0.849        0.976
##    49     71       1    0.897  0.0344        0.833        0.967
## 
##                 CDC_GRUPPO=Chirurgico, QUALIF_GRUPPO=Inferm. 
##  time n.risk n.event survival std.err lower 95% CI upper 95% CI
##     0    578        2    0.997 0.00244        0.992        1.000
##     4    576        1    0.995 0.00299        0.989        1.000
##     7    575        1    0.993 0.00345        0.986        1.000
##     9    574        1    0.991 0.00385        0.984        0.999
##    10    573        1    0.990 0.00422        0.981        0.998
##    11    572       11    0.971 0.00703        0.957        0.984
##    12    561        1    0.969 0.00723        0.955        0.983
##    13    560        1    0.967 0.00742        0.953        0.982
##    14    559        3    0.962 0.00796        0.946        0.978
##    15    556        1    0.960 0.00813        0.944        0.976
##    16    555        1    0.958 0.00830        0.942        0.975
```

```
##    17    554        1    0.957 0.00846        0.940        0.973
##    18    553        1    0.955 0.00862        0.938        0.972
##    21    552        5    0.946 0.00937        0.928        0.965
##    22    547        3    0.941 0.00979        0.922        0.961
##    23    544        8    0.927 0.01080        0.906        0.949
##    24    536        5    0.919 0.01137        0.897        0.941
##    25    531        2    0.915 0.01159        0.893        0.938
##    28    529        4    0.908 0.01200        0.885        0.932
##    29    525        3    0.903 0.01230        0.879        0.928
##    30    522        2    0.900 0.01250        0.875        0.924
##    31    520        3    0.894 0.01278        0.870        0.920
##    35    517        2    0.891 0.01296        0.866        0.917
##    36    515        1    0.889 0.01305        0.864        0.915
##    38    514        8    0.875 0.01374        0.849        0.903
##    40    506        2    0.872 0.01390        0.845        0.900
##    42    504        2    0.869 0.01406        0.841        0.897
##    43    502        4    0.862 0.01436        0.834        0.890
##    49    498        2    0.858 0.01451        0.830        0.887
##    50    496        1    0.856 0.01459        0.828        0.885
##    51    495        2    0.853 0.01473        0.825        0.882
##    59    252        4    0.839 0.01598        0.809        0.871
## 
##                CDC_GRUPPO=Chirurgico, QUALIF_GRUPPO=Prof. San. 
##  time n.risk n.event survival std.err lower 95% CI upper 95% CI
##    11    248        1    0.996 0.00402        0.988        1.000
##    14    247        1    0.992 0.00568        0.981        1.000
##    15    246        2    0.984 0.00800        0.968        1.000
##    17    244        1    0.980 0.00893        0.963        0.997
##    21    243        1    0.976 0.00976        0.957        0.995
##    22    242        3    0.964 0.01188        0.941        0.987
##    23    239        1    0.960 0.01249        0.936        0.984
##    25    238        3    0.948 0.01415        0.920        0.976
##    28    235        1    0.944 0.01466        0.915        0.973
##    29    234        1    0.940 0.01514        0.910        0.970
##    31    233        2    0.931 0.01605        0.901        0.963
##    36    231        1    0.927 0.01647        0.896        0.960
##    38    230        3    0.915 0.01768        0.881        0.951
##    40    227        1    0.911 0.01805        0.877        0.947
##    57    124        1    0.904 0.01935        0.867        0.943
## 
##                CDC_GRUPPO=Medico, QUALIF_GRUPPO=Inferm. 
##  time n.risk n.event survival std.err lower 95% CI upper 95% CI
##     4    810        1    0.999 0.00123        0.996        1.000
##     8    809        2    0.996 0.00213        0.992        1.000
##     9    807        1    0.995 0.00246        0.990        1.000
##    10    806        5    0.989 0.00368        0.982        0.996
##    11    801        3    0.985 0.00424        0.977        0.994
##    12    798        1    0.984 0.00442        0.975        0.993
##    14    797        2    0.981 0.00474        0.972        0.991
##    15    795        4    0.977 0.00532        0.966        0.987
```

```
##    16    791       1    0.975 0.00545        0.965        0.986
##    17    790       8    0.965 0.00642        0.953        0.978
##    18    782       2    0.963 0.00664        0.950        0.976
##    19    780       1    0.962 0.00674        0.949        0.975
##    21    779       3    0.958 0.00705        0.944        0.972
##    22    776       8    0.948 0.00779        0.933        0.964
##    23    768       4    0.943 0.00813        0.927        0.959
##    24    764       9    0.932 0.00884        0.915        0.950
##    25    755       5    0.926 0.00920        0.908        0.944
##    28    750       7    0.917 0.00968        0.899        0.936
##    29    743       6    0.910 0.01006        0.890        0.930
##    31    737      18    0.888 0.01110        0.866        0.910
##    32    719       1    0.886 0.01115        0.865        0.909
##    35    718       3    0.883 0.01131        0.861        0.905
##    36    715       7    0.874 0.01166        0.852        0.897
##    37    708       4    0.869 0.01185        0.846        0.893
##    38    704       6    0.862 0.01213        0.838        0.886
##    39    698       2    0.859 0.01222        0.836        0.884
##    40    696       3    0.856 0.01235        0.832        0.880
##    42    693       3    0.852 0.01248        0.828        0.877
##    43    690       4    0.847 0.01265        0.822        0.872
##    45    686       6    0.840 0.01290        0.815        0.865
##    46    680       2    0.837 0.01298        0.812        0.863
##    49    678       2    0.835 0.01306        0.809        0.861
##    50    676       3    0.831 0.01317        0.805        0.857
##    57    350       2    0.826 0.01352        0.800        0.853
## 
##                  CDC_GRUPPO=Medico, QUALIF_GRUPPO=Prof. San. 
##  time n.risk n.event survival std.err lower 95% CI upper 95% CI
##     7    291       1    0.997 0.00343        0.990        1.000
##     8    290       1    0.993 0.00484        0.984        1.000
##    14    289       1    0.990 0.00592        0.978        1.000
##    16    288       2    0.983 0.00762        0.968        0.998
##    17    286       1    0.979 0.00833        0.963        0.996
##    18    285       1    0.976 0.00898        0.958        0.994
##    22    284       1    0.973 0.00959        0.954        0.991
##    23    283       2    0.966 0.01068        0.945        0.987
##    24    281       2    0.959 0.01166        0.936        0.982
##    25    279       3    0.948 0.01296        0.923        0.974
##    28    276       1    0.945 0.01336        0.919        0.972
##    29    275       2    0.938 0.01412        0.911        0.966
##    30    273       2    0.931 0.01483        0.903        0.961
##    31    271       1    0.928 0.01517        0.899        0.958
##    32    270       1    0.924 0.01550        0.895        0.955
##    35    269       1    0.921 0.01582        0.890        0.952
##    36    268       3    0.911 0.01672        0.878        0.944
##    38    265       3    0.900 0.01756        0.867        0.935
##    39    262       2    0.893 0.01809        0.859        0.930
##    45    260       1    0.890 0.01834        0.855        0.927
##    49    259       1    0.887 0.01859        0.851        0.924
```

```
##    50    258        2    0.880 0.01907        0.843        0.918
##    58    132        1    0.873 0.02005        0.835        0.913
## 
##                   CDC_GRUPPO=Servizi, QUALIF_GRUPPO=Inferm. 
##  time n.risk n.event survival std.err lower 95% CI upper 95% CI
##    17    128        1    0.992 0.00778        0.977        1.000
##    18    127        2    0.977 0.01337        0.951        1.000
##    24    125        1    0.969 0.01538        0.939        0.999
##    25    124        2    0.953 0.01868        0.917        0.990
##    29    122        1    0.945 0.02010        0.907        0.986
##    30    121        2    0.930 0.02260        0.886        0.975
##    31    119        1    0.922 0.02372        0.877        0.970
##    36    118        1    0.914 0.02477        0.867        0.964
##    39    117        3    0.891 0.02759        0.838        0.946
##    42    114        1    0.883 0.02843        0.829        0.940
##    50    113        2    0.867 0.03000        0.810        0.928
## 
##                   CDC_GRUPPO=Servizi, QUALIF_GRUPPO=Prof. San. 
##  time n.risk n.event survival std.err lower 95% CI upper 95% CI
##     8    201        1    0.995 0.00496        0.985        1.000
##     9    200        3    0.980 0.00985        0.961        1.000
##    10    197        1    0.975 0.01099        0.954        0.997
##    11    196        5    0.950 0.01534        0.921        0.981
##    14    191        1    0.945 0.01604        0.914        0.977
##    15    190        1    0.940 0.01671        0.908        0.974
##    17    189        2    0.930 0.01796        0.896        0.966
##    18    187        2    0.920 0.01909        0.884        0.959
##    21    185        1    0.915 0.01963        0.878        0.955
##    22    184        2    0.905 0.02064        0.866        0.947
##    24    182        2    0.896 0.02158        0.854        0.939
##    25    180        5    0.871 0.02367        0.825        0.918
##    28    175        4    0.851 0.02513        0.803        0.901
##    29    171        1    0.846 0.02547        0.797        0.897
##    31    170        4    0.826 0.02675        0.775        0.880
##    32    166        4    0.806 0.02789        0.753        0.863
##    38    162        2    0.796 0.02842        0.742        0.854
##    39    160        2    0.786 0.02892        0.731        0.845
##    40    158        1    0.781 0.02917        0.726        0.840
##    46    157        1    0.776 0.02940
```
