# Supplementary material for: Influenza Vaccination of Nurses and Other Health Care Workers in Different Occupational Settings: A Classic and AI Mixed Approach for Time-to-Event Data
Source: Nurs Rep. 2025 Mar 3;15(3):87. doi: 10.3390/nursrep15030087 (PMC11944323; doi:10.3390/nursrep15030087)
Supplement: Supplementary file 1 [file nursrep-15-00087-s001.zip › SuppMat_S2.pdf]

SUPPLEMENTARY MATERIALS S2

Article

Influenza Vaccination of Nurses and Other Health Care Workers in Different Occupational Settings: a Classic and AI Mixed Approach for Time-to Event Data

Matteo Ratti, Riccardo Rescinito, Domenico Gigante, Alberto Lontano, and Massimiliano Panella

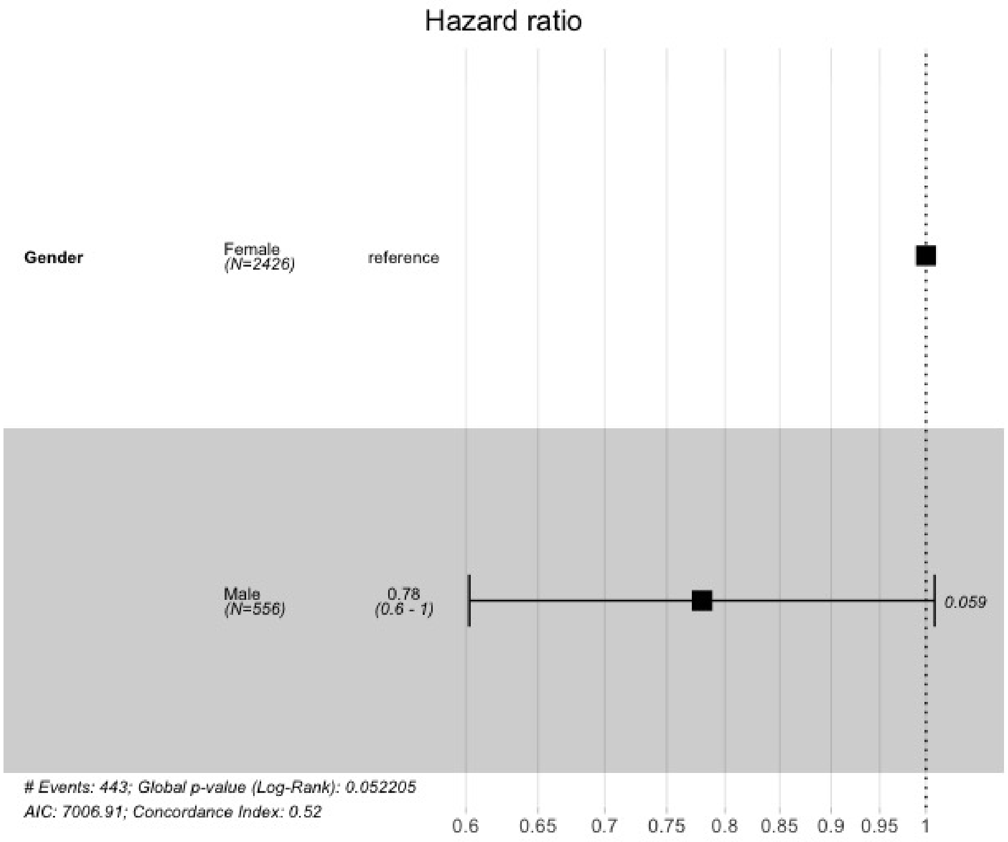

Figure S2-1 - Gender univariate cox PH model result

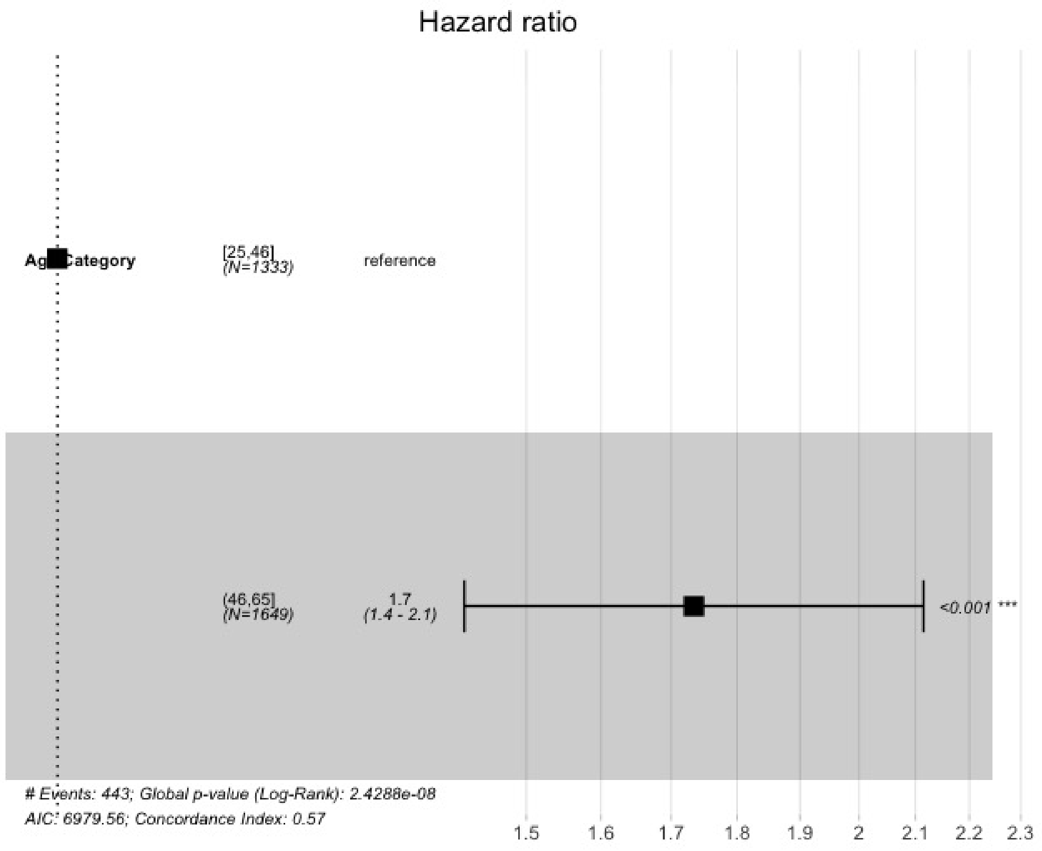

Figure S2-2 – Age category univariate cox PH model result

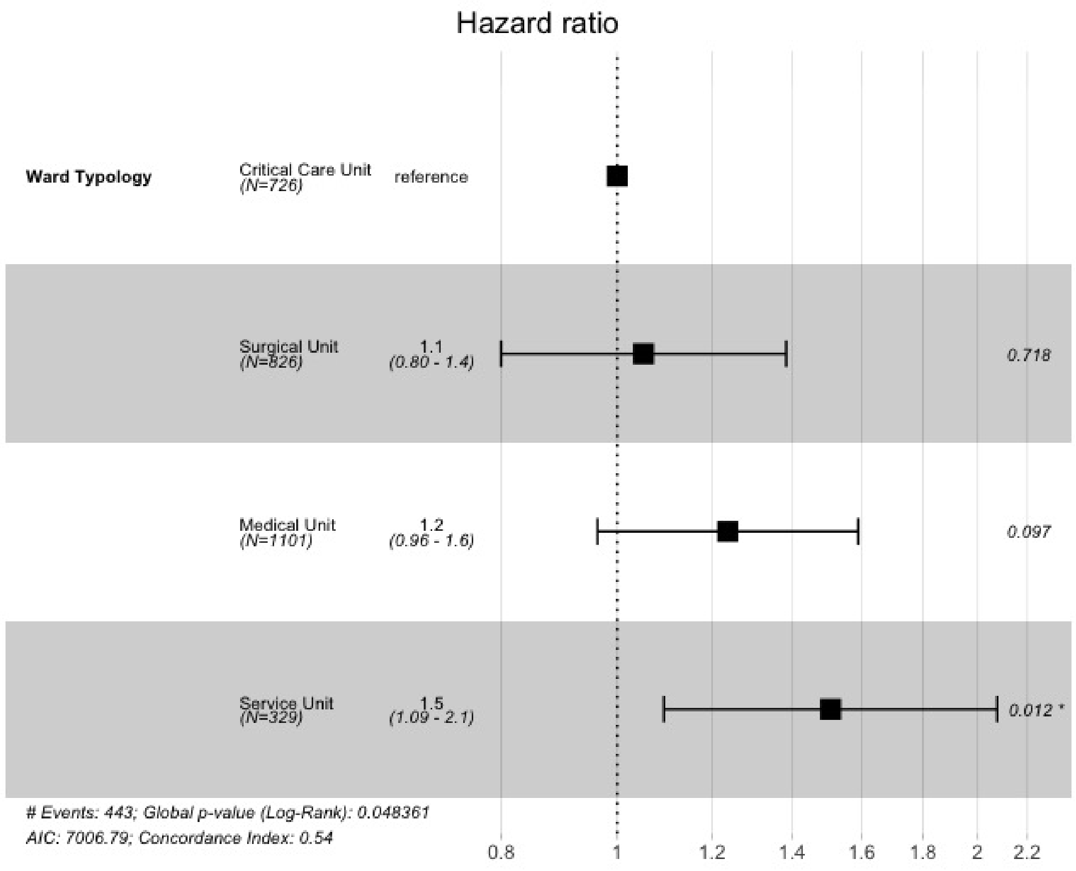

Figure S2-3 – Ward Typology univariate cox PH model result

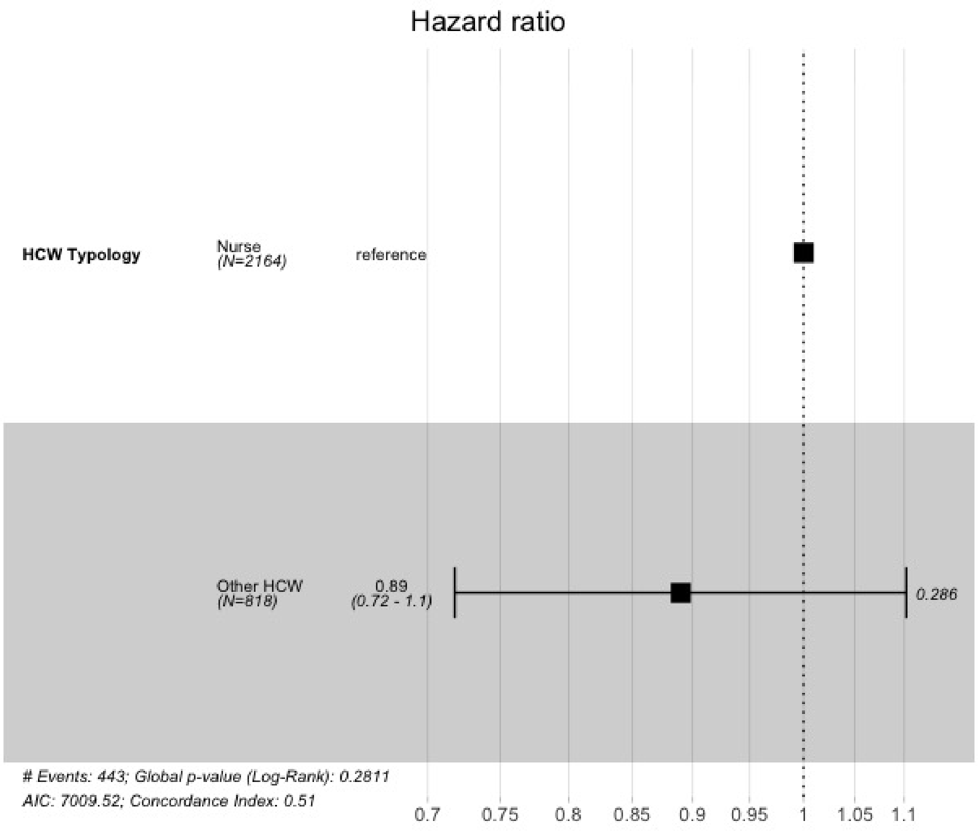

Figure S2-4 – HCW Typology univariate cox PH model result

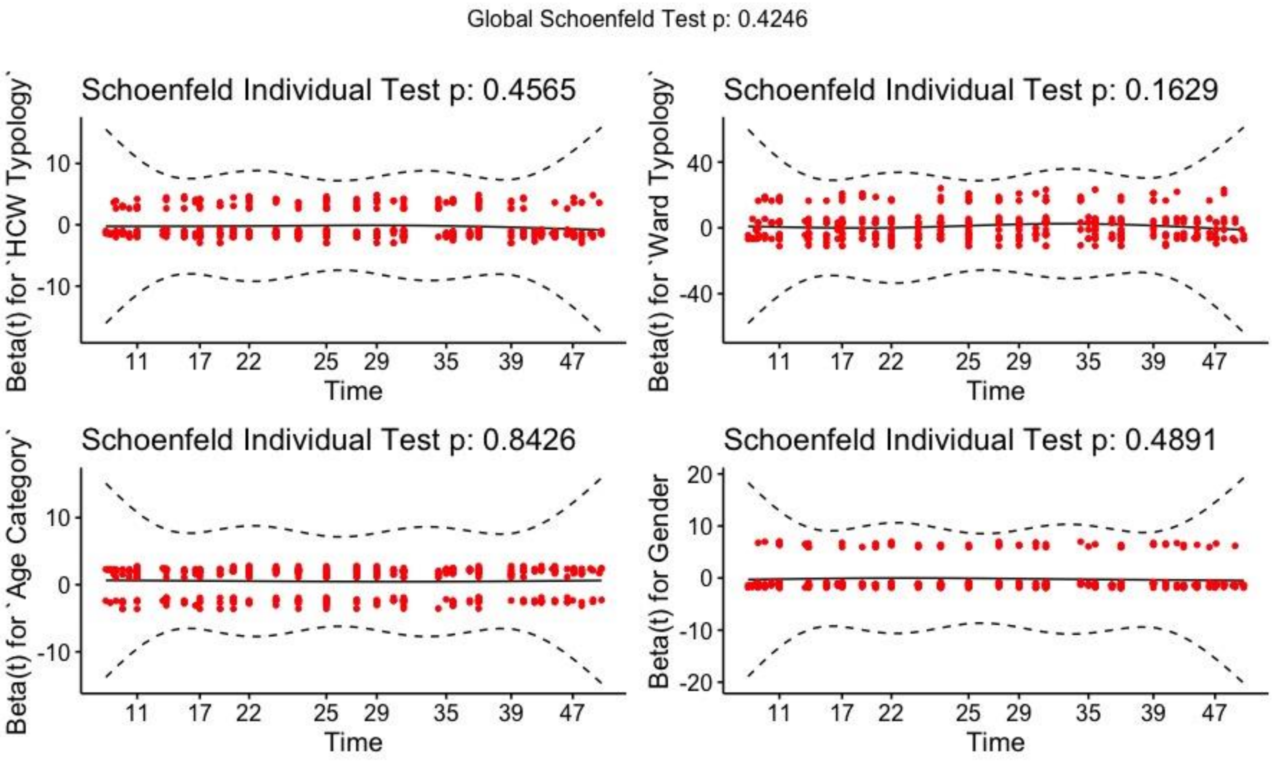

Figure S2-5 – Scaled Shoenfeld residuals for the multi variable cox model fit.

```
Ranger result

Call:
 ranger(Surv(TIME_F, STATUS_F) ~ WardTypology + AgeCategory +      HCWTypology + Gender, data = as.data.frame(db_randomF))

Type:                             Survival
Number of trees:                  500
Sample size:                      2982
Number of independent variables:  4
Mtry:                             2
Target node size:                 3
Variable importance mode:         none
Splitrule:                        logrank
Number of unique death times:     44
OOB prediction error (1-C):       0.4358655
```

Figure S2-6 - Results of the random forest survival model fit with parameter values
